# Faecal immunochemical testing in bowel cancer screening: Estimating outcomes for different diagnostic policies

**DOI:** 10.1177/0969141320980501

**Published:** 2020-12-20

**Authors:** Shuping J Li, Linda D Sharples, Sally C Benton, Oleg Blyuss, Christopher Mathews, Peter Sasieni, Stephen W Duffy

**Affiliations:** 1 Wolfson Institute of Preventive Medicine, Queen Mary University of London, London, UK; 2 Department of Medical Statistics, London School of Hygiene and Tropical Medicine, London, UK; 3 NHS Bowel Cancer Screening Programme, Royal County Hospital NHS Foundation Trust, Guildford, Surrey, UK; 4 School of Physics, Astronomy and Mathematics, University of Hertfordshire, Hertfordshire, UK; 5 Department of Paediatrics and Paediatric Infectious Diseases, Sechenov First Moscow State Medical University, Moscow, Russia; 6 School of Cancer and Pharmaceutical Sciences, King’s College London, London, UK

**Keywords:** Bowel cancer, colorectal cancer, screening policies, faecal immunochemical test, FIT thresholds, iFOBT

## Abstract

**Objectives:**

The National Health Service Bowel Cancer Screening Programme (NHS BCSP) in England has replaced guaiac faecal occult blood testing by faecal immunochemical testing (FIT). There is interest in fully exploiting FIT measures to improve bowel cancer (CRC) screening strategies. In this paper, we estimate the relationship of the quantitative haemoglobin concentration provided by FIT in faecal samples with underlying pathology. From this we estimate thresholds required for given levels of sensitivity to CRC and high-risk adenomas (HRA).

**Methods:**

Data were collected from a pilot study of FIT in England in 2014, in which 27,238 participants completed a FIT. Those with a faecal haemoglobin concentration (f-Hb) of at least 20 µg/g were referred for further investigation, usually colonoscopy. Truncated regression models were used to explore the relationship between bowel pathology and FIT results. Regression results were applied to estimate sensitivity to different abnormalities for a number of thresholds.

**Results:**

Participants with CRC and HRA had significantly higher f-Hb, and this remained unchanged after adjusting for age and sex. While a threshold of 20 μg/g was estimated to capture 82.2% of CRC and 64.0% of HRA, this would refer 7.8% of participants for colonoscopy. The current programme threshold used in England of 120 μg/g was estimated to identify 47.8% of CRC and 25.0% of HRA.

**Conclusions:**

Under the current diagnostic policy of dichotomising FIT results, a very low threshold would be required to achieve high sensitivity to CRC and HRA, which would place further strain on colonoscopy resources. The NHS BCSP in England might benefit from a diagnostic policy that makes greater use of the quantitative nature of FIT.

## Introduction

Bowel cancer (colorectal cancer, CRC) is the second most common cause of cancer death in the UK, accounting for 10% of all cancer deaths in 2017.^1^ Between 2015 and 2017, there were around 16,300 CRC deaths in the UK every year, equivalent to 45 deaths every day.^1^ In order to reduce mortality and incidence of CRC, the National Health Service Bowel Cancer Screening Programme (NHS BCSP) in England offers tests for the presence of occult blood in faeces free of charge every two years for men and women aged 60–74 years (inclusive).

Up to 7 June 2019, the guaiac faecal occult blood test (gFOBT) was the method used and this gives a binary result (positive or negative) for each sample.^2,3^ It requires two faecal samples from each of three separate bowel motions to attain satisfactory sensitivity. In contrast, the faecal immunochemical test (FIT) gives a quantitative result in the form of micrograms of haemoglobin per gram of faeces (μg/g), and requires only a single sample. In 2014 the NHS BCSP in England performed a pilot study to examine the acceptability and diagnostic performance of FIT in two of the five regional hubs managing the established screening programme in England.

The main analysis of the FIT pilot study by Moss et al.^3^ assessed the effect of varying faecal haemoglobin concentration (f-Hb) threshold on detection rate of CRC and advanced adenomas (high-risk and intermediate-risk adenomas combined, as defined by Moss et al: see the Pathology section for definitions), and on the colonoscopy rate. This information was very useful for informing the national programme, but because those with f-Hb less than 20 μg/g did not receive further investigation, it did not estimate the numbers of abnormalities missed for a given threshold. Also, it did not consider the relationship in the direction of causality: it is the abnormalities that cause bleeding, and therefore the FIT result. This paper aims to complement the previous results by:

1. Exploring the relationship between FIT results and bowel pathology using truncated regression, in both a univariate and multiple regression model, with demographic factors including age, sex and area-based socioeconomic status; and
2. Using these results to estimate proportions of bowel abnormalities the screening programme would fail to diagnose at different FIT thresholds (false negative rates);
3. Generating hypotheses for fuller exploitation of quantitative FIT measures.

## Methods

### Bowel cancer screening programme in England

**Figure 1.**
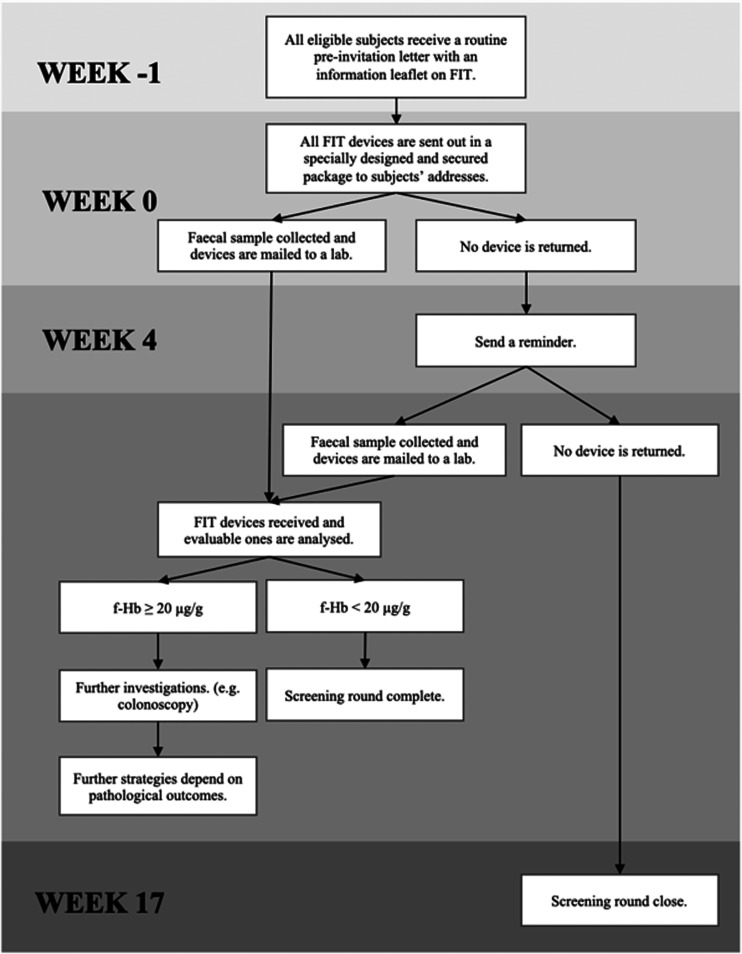
Time frame of the English Faecal Immunochemical Test (FIT) Pilot Study 2014, from pre-invitation period to the end of the screening round.

The NHS BCSP in England started to adopt FIT in June 2019.^2^ Currently, the policy is to have a single threshold indicating further diagnostic workup for those at or above the threshold, or return to routine screening for those below. The diagnostic performance of FIT for detecting CRC and adenomas depends on the threshold defined for positivity. The programme uses a threshold of 120 μg/g for referral for further investigation (Figure 1).

### Study population

The 2014 FIT pilot study drew samples from the routine screening population invited by two of the five English BCSP Hubs (the Midlands and North West Hub and the Southern Hub). The study protocol pseudo-randomly assigned every 28th consecutive invitee to receive a FIT instead of a gFOBT kit.^3^ Those who were offered FIT will be referred to as invitees below, and those who gave valid FIT results will be referred to as participants. There were 40,928 invitees aged 59–75 (inclusive) years old, and 27,238 participants (14,404 women and 12,834 men). Only those 2133 participants with a positive f-Hb, defined as at least 20 μg/g, were invited for further investigation (usually colonoscopy). At the end of the pilot study, 1825 participants had a definitive pathology outcome. These are referred to as complete cases below.

The dataset used in this paper was extracted from BCSS (Bowel Cancer Screening System) with reference ODR_1819_103. It has been substantially updated and cleaned since the previous publication, in particular including FIT results which became available after the previous publication was written, and so will not have exactly the same numbers as previously reported.^3–5^ Compared to the previous paper, we therefore report on two fewer invitees (40,928 vs. 40,930), 71 more participants with valid FIT results (27,238 vs. 27,167), six more positive tests (2133 vs. 2127) and one more colonoscopy result (1825 vs. 1824).

### Pathology

After a colonoscopy, pathologists examined removed tissues (if any) and classified those according to the recommendations from The British Society of Gastroenterology (BSG).^6^ In this paper, we re-categorised pathology outcomes defined as follows:

1. Low-risk adenomas (LRA), if removed tissues contained 1–2 adenomas and were both small (less than 1 cm in diameter);
2. Intermediate-risk adenomas (IRA), if removed tissues contained 3–4 small adenomas or 1–2 adenomas of which at least one has diameter greater than or equal to 1 cm;
3. High-risk adenomas (HRA), if removed tissues contained at least five small adenomas or three or more adenomas at least one of which is greater or equal to 1 cm in diameter;
4. Cancer (CRC) if removed tissues had characteristic of malignancy;
5. Other abnormality, to include all other unclassified abnormalities; and
6. No abnormality, if removed tissues contained no abnormalities.

### Statistical analysis

In order to estimate the potential impact of deprivation on FIT results, we used the Index of Multiple Deprivation (IMD), which is the official and the most widely used measure of deprivation in England.^7^

FIT results less than 4 μg/g were recoded to 1 μg/g (rather than zero due to the natural logarithm transformation later) and are referred to as undetectable f-Hb.^8^ The limit of detection is 4 μg/g; that is FIT analysers (OC-Sensor DIANA, Eiken Chemical Co., Ltd, Japan) cannot distinguish samples with f-Hb lower than this from samples with no f-Hb.

We carried out univariable and multivariable regression analyses with the quantitative FIT measure (f-Hb) as the dependent and pathology outcomes as independent variables. We compared proportions of participants with undetectable f-Hb in their samples among demographic and screening episode characteristics using logistic regression.

We used truncated regression since there were no pathology data on participants with f-Hb less than 20 μg/g.^9^ As a result of the skewed distribution of f-Hb, we used the natural logarithm of f-Hb as the dependent variable. Regression coefficients were then transformed back to the original scale to provide the ratio of geometric mean f-Hb for each pathology, relative to no abnormality pathology. Likelihood ratio tests were used to select the best model, with Wald tests helping to identify significant categories (*p* < 0.001) within a variable. Given previous observed sex- and age-specific differences,^3–5^ the multivariable model included these variables.

We also fitted truncated regression models with only constant terms and no predictor variables, restricting analysis to each pathology category separately. These are referred to as univariable regression models below. From these results, we estimated the distributions of f-Hb for different pathology outcomes. Using these, we then estimated the proportions of abnormalities captured by different f-Hb thresholds, and by implication, the proportions missed for those thresholds. We also considered the problem from the opposite angle, calculating the thresholds required for given levels of sensitivity to CRC and HRA.

All analyses were carried out in StataMP version 15.1 on a Windows 8 platform.

## Results

Table 1 shows the demographics and screening episode of the 40,928 invitees, and of the subpopulation of 27,238 participants who provided a valid FIT sample; the latter provides the dataset for analysis of the association between pathology and f-Hb. This subpopulation was characterised by relatively high proportions of non-deprived invitees (more than 50% were in the two least deprived IMD quintiles) and previous participants in CRC screening (75.1%).

**Table 1.** Baseline characteristics of invitees and participants.

	All invited (*n* = 40,928)		Valid FIT (*n* = 27,238)	
	*N*	%	*N*	%
Hub				
Southern	21,640	52.9	14,743	54.1
Midlands and North West	19,288	47.1	12,495	45.9
Sex				
Female	21,064	51.5	14,404	52.9
Male	19,864	48.5	12,834	47.1
Age group in years				
59–64	17,428	42.6	11,154	41.0
65–69	14,037	34.3	9685	35.6
70–75	9463	23.1	6399	23.5
IMD quintile				
IMD 1 (most deprived)	5775	14.1	3016	11.1
IMD 2	6560	16.0	4081	15.0
IMD 3	8676	21.2	5883	21.6
IMD 4	9554	23.3	6686	24.6
IMD 5 (least deprived)	10,357	25.3	7568	27.8
IMD n/k^a^	6	0.01	4	0.01
Screening episode				
Previous responders	22,737	55.6	20,465	75.1
First-time invitees	6453	15.8	3962	14.6
Previous non-responders	11,738	28.7	2811	10.3
Overall	40,928	100.0	27,238	66.6

Note: IMD 1 to IMD 5 is the scale from the most deprived to the least deprived. FIT: Faecal immunochemical test; IMD: index of multiple deprivation.

^a^Participants where postcode could not be linked to layer super output areas (LSOA).

Table 2 summarises categories of observed f-Hb by demographic factors and screening episodes. The table gives the numbers and proportions with undetectable f-Hb, detectable f-Hb and positive f-Hb (f-Hb ≥ 20 μg/g), for the latter two subpopulations also giving the geometric mean and empirical 80% ranges of f-Hb. Large proportions of participants had undetectable f-Hb in all subgroups. There were significantly lower proportions with undetectable f-Hb (i.e. higher proportions with some evidence of bleeding) in the Midlands and North Western Hub participants, in males, in older participants, in more deprived populations, and in previous non-responders (*P* < 0.001 in all cases).

**Table 2.** Frequencies (proportions) of participants with undetectable f-Hb, and frequencies (proportions), geometric means and 80% empirical ranges for participants with detectable and positive f-Hb (µg/g), stratified by demographic characteristics and screening episodes.

	Undetectable f-Hb^a^ (<4 µg/g)		Detectable f-Hb^a^ (≥4 µg/g)				Positive f-Hb (≥20 µg/g)			
	*N*	%	*N*	%	Mean^b^	80% PR^c^	*N*	%	Mean^b^	80% PR^c^
Hub										
Southern	11,965	81.2	2778	18.8	19	5–126	1049	7.1	78	24–405
Midlands and North West	9468	75.8	3027	24.2	18	5–113	1084	8.7	78	25–389
Sex										
Female	11,553	80.2	2851	19.8	16	5–90	947	6.6	69	24–300
Male	9880	77.0	2954	23.0	21	5–153	1186	9.2	86	25–481
Age group, years										
59–64	9012	80.8	2142	19.2	18	5–120	771	6.9	78	25–389
65–69	7619	78.7	2066	21.3	18	5–120	747	7.7	80	25–383
70–75	4802	75.0	1597	25.0	19	5–113	615	9.6	76	24–401
IMD quintile^d^										
IMD 1 (most deprived)	2199	72.9	817	27.1	20	5–123	324	10.7	75	24–359
IMD 2	3087	75.6	994	24.4	20	5–122	392	9.6	78	25–432
IMD 3	4657	79.2	1226	20.8	18	5–117	435	7.4	80	25–394
IMD 4	5314	79.5	1372	20.5	18	5–118	504	7.5	79	25–405
IMD 5 (least deprived)	6172	81.6	1396	18.4	17	5–111	478	6.3	78	24–416
IMD n/k^e^	4	.	0	.	.	.	0	.	.	.
Screening episode (*n*, %)										
Previous responders	16,109	78.7	4356	21.3	18	5–111	1592	7.8	75	24–367
First-time invitees	3234	81.6	728	18.4	17	5–112	248	6.3	82	25–522
Previous non-responders	2090	74.4	721	25.6	22	5–175	293	10.4	91	25–450
Overall	21,433	78.7	5805	21.3	18	5–118	2,133	8	78	25–394

^a^f-Hb, faecal haemoglobin concentration (µg/g).

^b^Geometric mean. ^c^80% percentile range (PR) is the 10th and the 90th percentile observed.

^d^IMD, index of multiple deprivation. IMD 1 to IMD 5 is the scale from the most deprived to the least deprived.

^e^Participants whose postcode could not be linked to lower layer super output areas (LSOA).

Among positive cases, the overall geometric mean f-Hb was 78 μg/g, compared with 18 μg/g for participants with a detectable f-Hb. In the whole study population, FIT results varied by screening hubs, sex, age groups and deprivation index. Average f-Hb was similar between participants in the Midlands and North West Hub and those in the Southern Hub, although the latter hub had a higher proportion with undetectable f-Hb. Male participants, older participants and more deprived participants all had a higher geometric mean f-Hb. The age effect was largely due to lower numbers with undetectable f-Hb in the oldest age group. Although previous non-responders had a higher geometric mean f-Hb than either first-time invitees or previous responders, this was not statistically significant (although the previous non-respondents had a significantly lower proportion of undetectable f-Hb).

For those with f-Hb of at least 20 μg/g, geometric means were similar in all strata, with the exception of sex: males had a much higher geometric mean f-Hb in both the whole population and among the positive cases only. This suggests that most of the other demographic differences are predominantly driven by the proportions of undetectable f-Hb. However, although in this group as a whole there was no clear trend in f-Hb with age, there was a greater tendency for older subjects with no CRC or adenoma to have f-Hb of 20 μg/g or more: the proportions were 2.8%, 3.1% and 3.3% for age groups 59–64, 65–69 and 70–75 years, respectively (*p* = 0.045).

### Understanding the relationship between FIT-detected f-Hb and pathology

As noted in the Methods section, the truncated regression was carried out on the 1825 complete cases. The final multiple regression model (supplied in table S1) adjusted for age and sex suggests that participants with CRC and HRA had significantly higher f-Hb (*p* < 0.001). After controlling for age and sex, participants who had CRC and HRA, respectively, had log(f-Hb) approximately 3.08 higher and 1.53 higher than those with no abnormality. Back-transforming to the original scale, a participant with CRC was estimated to have f-Hb 22 times that of a participant of the same age and sex but with no abnormal pathology (on average). After adjusting for age and sex, the f-Hb of participants who had LRA or other abnormality were not statistically significant different from participants who had no abnormality pathology (Wald tests, *p* = 0.855 and *p* = 0.791).

**Figure 2.**
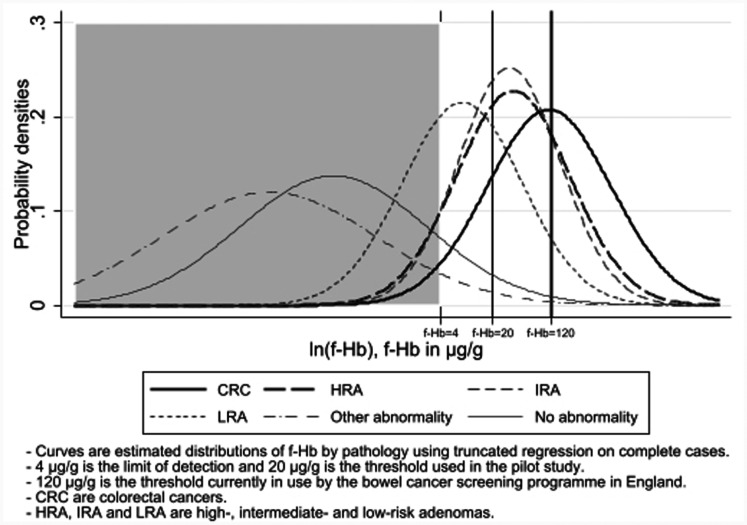
Distribution of f-Hb estimated by truncated regression with pathology on 1825 complete cases.

Table 3 gives the empirical geometric mean, and 10th to 90th percentile ranges (referred to below as 80% ranges) of f-Hb for each pathology and the corresponding estimated 80% ranges of the distribution of f-Hb, calculated from the univariable truncated regression models. We calculated 80% ranges rather than 95% ranges (used in laboratory quality control), as the latter were so wide as to be uninformative as to the concentrations characterising the central bulk of the population. If the model is a good fit, we expect 80% of cases to have observed f-Hb within the 80% range estimated from the distribution. We note that whilst the average f-Hb (geometric means) is strongly associated with pathology, the 80% ranges are very wide, particularly for CRC and HRA. To illustrate the overlap, Figure 2 shows the estimated distributions of f-Hb by pathology on the same graph. There is a reasonable separation between CRC and no abnormality and other abnormality, and a rather poorer separation of adenoma pathology from no abnormality and other abnormality.

**Table 3.** Summary of faecal haemoglobin concentration (f-Hb) by pathology, with counts and corresponding proportion in colonoscopy; geometric mean and 80% empirical percentile ranges, and 80% normal ranges estimated by truncated regression on 1825 complete cases.

	*N*	% of colonoscopy^a^	f-Hb (μg/g)		
			Geometric Mean	80% range^b^	80% estimated range^c^
Colorectal cancer	74	4.1	115	38–2178	10–1339
High-risk adenoma	216	11.8	38	26–723	4–356
Intermediate-risk adenoma	261	14.3	33	28–419	4–252
Low-risk adenoma	470	25.8	8	25–221	1–84
Other abnormality	538	29.5	0	24–312	0–1
No abnormality	266	14.6	0	24–271	0–6

80% range = geometric mean ± 1.1.28standard deviation.

^a^Participants with colorectal cancer as a proportion of those underwent colonoscopy. For example, 4.1% = 74/1825.

^b^80% range is the 10th and the 90th percentile observed.

^c^80% estimated range are estimations from univariable truncated regression using ln(f-Hb) as the dependent variable.

**Figure 3.**
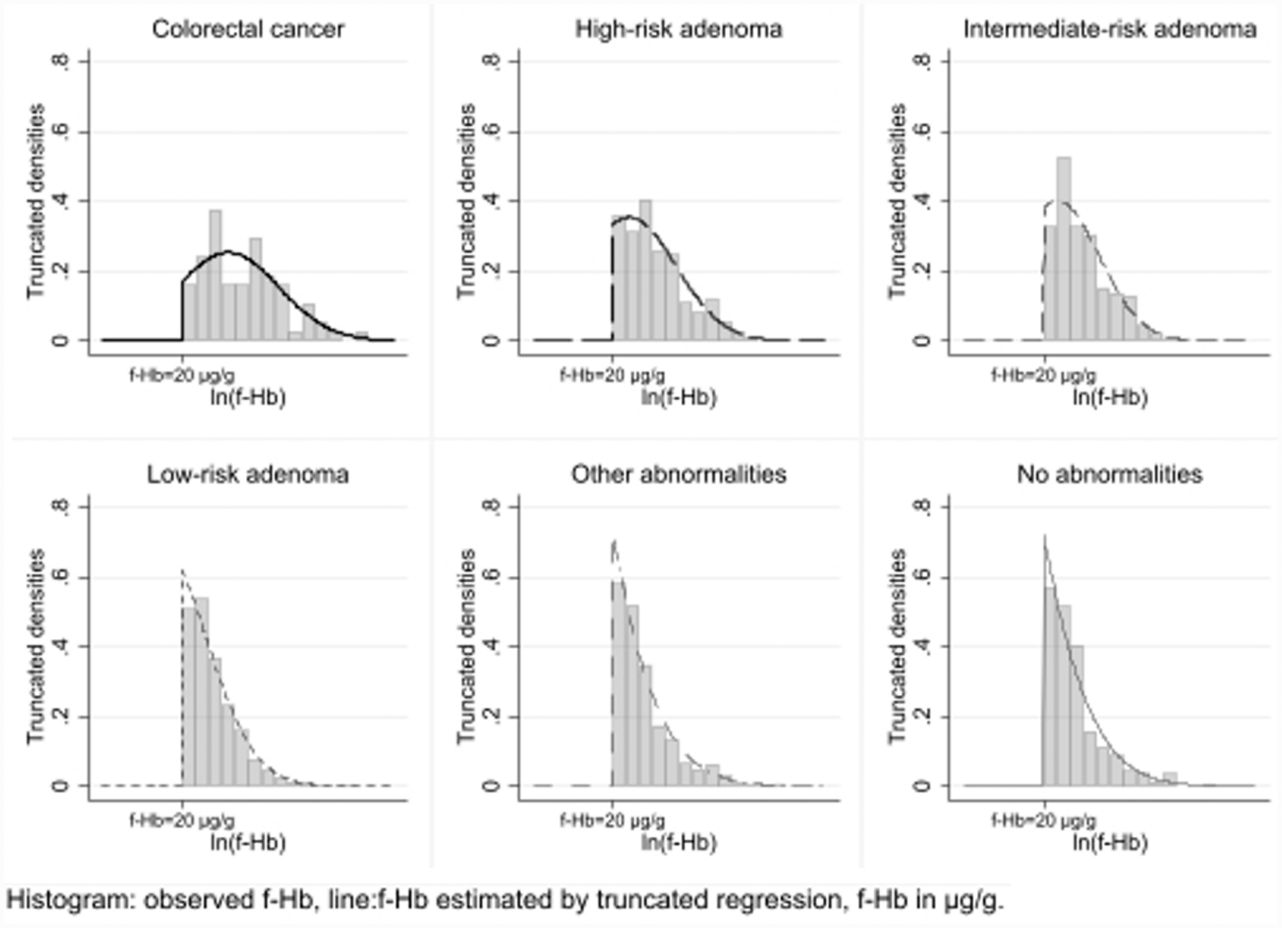
Distributions of observed f-Hb on the log scale for f-Hb at least 20 μg/g and estimated FIT distributions from truncated regression models, for each pathology, with mean, standard deviation and estimated percentage observed. There were 1825 complete cases only.

Figure 3 shows the estimated distribution of f-Hb within each pathology with histograms corresponding to observed f-Hb that are equal to or above 20 μg/g. Average f-Hb is very low for most categories, with a high degree of variation. For example, CRC having a mean and SD of 4.74 and 1.92, respectively, on the logarithmic scale corresponds to an 80% range in the linear scale of around 10–1339 μg/g.

### Using regression results to estimate sensitivity of the programme to different bowel pathologies

Using the univariable truncated regression results, Table 4 shows the estimated sensitivity of FIT to CRC and HRA, and the percentage of participants who would be recalled for a number of thresholds. For example, a low threshold such as 20 µg/g results in high sensitivity for detection of CRC and moderate sensitivity for HRA, but would require almost 7.8% of participants to undergo colonoscopy. Note that specificity cannot be calculated since negative FIT did not result in further investigation.

**Table 4.** Sensitivities to CRC and HRA by f-Hb thresholds and observed numbers and proportions above these thresholds.

	Sensitivity for pathology (number and percentage) above threshold							
	CRC		HRA		No/other abnormality^a^		All participants	
Threshold (μg/g)	Number	%	Number	%	Number	%	Number	%
4	(86)	95.6	(303)	90.2	(4051)	16.5	5805	21.3
10	(81)	90.0	(261)	77.7	(2071)	8.4	3423	12.6
20	74	82.2	216	64.3	1112	4.5	2133	7.8
40	66	73.3	168	50.0	703	2.9	1421	5.2
80^b^	53	58.9	108	32.1	375	1.5	802	2.9
120^b^	43	47.8	84	25.0	269	1.1	576	2.1
150^b^	40	44.4	70	20.8	228	0.9	483	1.8
180	36	40.0	59	17.6	195	0.8	411	1.5

Note: Numbers for thresholds 4 and 10 (in brackets) are estimated from univariable truncated regression model on 1825 complete cases. All others observed from all 27,238 participants in the pilot study.

^a^‘No/other abnormality’ are calculated by subtraction from observed totals.

^b^80, 120 and 150 μg/g are thresholds chosen for the national programmes in Scotland, England and Wales, so included here.

Table 5 shows estimated prevalence in different intervals, with sensitivities estimated from the model. Consequently, these differ from those presented in Table 4, which were based on observed frequencies. However, the differences are very small, indicating that the model fits rather well to the CRC and HRA data (a more detailed comparison between estimated and observed frequencies is presented in Table S2). The table also gives the sensitivity of the lower point of each interval as a threshold for further investigation. That is, a threshold of 20 μg/g would confer 82.2% sensitivity to CRC and 64.0% sensitivity to HRA. The table indicates that the current threshold of 120 μg/g has a poor sensitivity for both CRC and HRA, only correctly identifying 48.9% and 25.6%, respectively (Table 5). Further, a very low FIT threshold (40 μg/g) is required in order to detect 71.1% of CRC and 48.2% of HRA (Table 5). The table also shows, for example, that for participants with f-Hb of 80–119 μg/g, 35.4 per 1000 (just under 4%) have CRC (more estimations on prevalence and sensitivity for all abnormalities by f-Hb are given in Tables S3–S5).

**Table 5.** Estimated prevalence of CRC and adenomas per 1000 participants and sensitivity to CRC and HRA by f-Hb categories; estimates derived from the regression results.

	Prevalence per 1000 participants					Sensitivity at a given threshold		
f-Hb category	CRC	HRA	IRA	LRA	HRA+	CRC	HRA	HRA+
<4	0.2	1.5	1.7	25.9	1.7	100.0%	100.0%	100.0%
4–9	2.1	17.6	23.5	125.5	19.7	95.6%	90.2%	91.3%
10–19	5.4	34.9	48.1	168.2	40.3	90.0%	77.7%	80.3%
20–39	14.0	73.0	101.1	252.8	87.1	82.2%	64.0%	68.1%
40–79	19.4	84.0	111.5	210.0	103.4	71.1%	48.2%	53.5%
80–119	35.4	115.0	146.0	230.1	150.4	57.8%	33.3%	38.5%
120–149	43.0	150.5	172.0	247.3	193.5	48.9%	25.6%	30.5%
150–179	41.7	125.0	166.7	222.2	166.7	44.4%	21.4%	26.3%
180+	90.0	153.3	143.6	167.9	243.3	41.1%	18.8%	23.5%

Note: Sensitivity estimated of the lower point of each interval as a threshold for further investigation. HRA+ are colorectal cancers and high-risk adenomas combined.

f-Hb: Faecal haemoglobin concentration (μg/g). CRC: colorectal cancer; HRA, IRA and LRA: high-, intermediate- and low-risk adenomas.

## Discussion

We analysed data from 27,238 FIT participants, and carried out truncated regression on the 1825 (complete cases) participants who underwent colonoscopy as a result of a FIT result (f-Hb) of 20 μg/g and above. We estimated the influence of demographic factors and colonoscopy findings on f-Hb, and calculated the expected results of different f-Hb thresholds in terms of both detected and missed CRC and adenomas.

The higher proportion with detectable levels in older participants is consistent with the results of Clark et al.^10^ In our data, the mean concentration among participants with positive results was relatively stable over age. This suggests that older participants have a greater tendency to bleeding and we might speculate that they do so regardless of presence or not of significant bowel abnormalities. Clark et al. suggest that this tendency may be a marker of systemic inflammation.^10^ Others have found that older participants have more false positive FIT results at the low threshold of 17 μg/g.^11^ In our data, with a threshold of 20 μg/g, there is also some evidence of this, with proportions of complete cases having no CRC or adenoma but with f-Hb of 20 μg/g or more being 2.8%, 3.1% and 3.3% for age groups 59–64, 65–69 and 70–75 years respectively (p = 0.045).

In contrast, amongst those with positive FIT results, males had a much higher geometric mean than females, and a much higher limit of the 80% range. Higher concentrations in males were also noted by Clark et al.^9^ and by Ribbing et al.^12^ Thus, the difference between males and females is driven in large measure by higher f-Hb of bleeding in those with positive f-Hb.

Since definitive pathology was only available for positive participants (f-Hb ≥20 µg/g), we used truncated regression methods to estimate the influence of bowel pathology on f-Hb and the sensitivity of different thresholds to CRC and HRA.

Using data from 1825 complete cases, we found that participants with CRC and HRA have considerably increased f-Hb, but that the variation among patients is very large. This has been observed by others.^13^ Despite distinguishing upper bounds in f-Hb amongst pathology of different risks, the large overlap at intermediate and low f-Hb imposes challenges under current dichotomised screening policies, in which participants in England with f-Hb at or above a single threshold (120 µg/g) are referred for colonoscopy and participants below that concentration receive their next screen two years later.

Our regression results indicate that the f-Hb threshold of 120 μg/g used in the NHS BCSP in England is likely to miss just over half of the CRC (51.1%) present at the time of sampling (Table 5). Ribbing et al. found considerably lower sensitivity at various thresholds for CRC and advanced adenoma (not the same as HRA; please refer to the paper for a precise definition) combined.^12^ In subjects with f-Hb of 80–119 μg/g, which would not trigger further investigation in the current programme in England, just under 4% had CRC (Table 5). This is higher than the 3% risk threshold for a two-week wait referral for suspected cancer in symptomatic subjects. To capture 80% of CRC and around 60% of HRA, a threshold of 22 μg/g is indicated by our results (Tables 4, 5 and S5). This is consistent with Whyte et al., who concluded that, in the absence of colonoscopy capacity issues, the most cost-effective FIT strategy would be a threshold of 20 μg/g.^14^ However, this would imply referring 7.5% of participants for colonoscopy (Table S5). Even prior to the COVID-19 crisis, colonoscopy capacity in England could not cope with this, and the capacity is likely to be even lower for the foreseeable future. Therefore, to maintain acceptable sensitivity to CRC and HRA, one might consider using the quantitative f-Hb more fully,^15^ with different actions for different f-Hb categories, for example:

- Undetectable f-Hb: delay next screen to three years;^16^
- Very low f-Hb: next screen in two years;^17^
- Low f-Hb: repeat screening test in three months to assess persistence of bleeding;^18^
- Medium f-Hb: flexible sigmoidoscopy to examine the lower part of the colon (distal), and remove any abnormalities found, followed by a further FIT to ascertain whether the cause of the bleeding has been removed;^19^
- High f-Hb: colonoscopy.

Note that we are not explicitly recommending exactly this strategy or these actions. This is simply an example of the approach one might take. More data is needed to ascertain the safety and effectiveness of such an approach, and to specify thresholds for different actions. Others have proposed varying strategies of f-Hb threshold and interscreening interval. For example, Haug et al. suggested a low threshold at first screen and a long interval to second.^20^ However, Digby et al. estimated that this would lead to non-negligible numbers of cases missed, and suggested as an alternative an interval determined on the basis of concentration at the first screen.^14^ Further research using this and other datasets will indicate the likely thresholds to define the above categories.

When truncated regression removes a majority of the data, estimates are less reliable.^9,21^ Thus, those with a ‘no abnormality’ or ‘other abnormality’ pathology would be overwhelmingly below the threshold of 20 μg/g, and therefore estimates for these would be less reliable. This is a limitation of the present study, and renders estimation of false positive results (no abnormality pathology with f-Hb above the threshold) uncertain. Estimation of false positive and false negative rates for thresholds below 20 μg/g remains a target for the future.

It should also be noted that the absolute f-Hb results reported here pertain specifically to the OC-Sensor DIANA analyser. While the observations of associations of demographic variables and pathology with concentrations are likely to be generalisable, exact numbers will not be.

Another limitation is that models fitted did not control for factors such as villous status and location of adenomas, which are known to influence f-Hb.^13,22^ These factors are also associated with risk of future CRC. Taking account of these is another target for the future.

## Conclusion

This analysis shows that the current threshold of 120 μg/g in the English NHS BCSP may only correctly identify half of CRC and a quarter of all HRA in the population. In order to achieve better detection rates of bowel abnormalities, while minimising the burden on endoscopy resources, the NHS BCSP might make use of the ability of FIT to provide quantitative results to develop a multi-threshold management strategy, thereby optimising clinical resources and patient outcomes.

## Supplemental Material

sj-pdf-1-msc-10.1177_0969141320980501 - Supplemental material for Faecal immunochemical testing in bowel cancer screening: Estimating outcomes for different diagnostic policies Click here for additional data file. Supplemental material, sj-pdf-1-msc-10.1177_0969141320980501 for Faecal immunochemical testing in bowel cancer screening: Estimating outcomes for different diagnostic policies by Shuping J Li, Linda D Sharples, Sally C Benton, Oleg Blyuss, Christopher Mathews, Peter Sasieni and Stephen W Duffy: on behalf of the CARE-MS I, CARE-MS II, and CAMMS03409 Investigators in Journal of Medical Screening

## References

[1] https://www.cancerresearchuk.org/health-professional/cancer-statistics/statistics-by-cancer-type/bowel-cancer (accessed 29 July 2020).

[2] https://www.england.nhs.uk/south/wp-content/uploads/sites/6/2019/06/fit-gp-briefing-sheet.pdf. (accessed 28 October 2020).

[3] Moss S Mathews C Day TJ, et al. Increased uptake and improved outcomes of bowel cancer screening with a faecal immunochemical test: results from a pilot study within the national screening programme in England. Gut 2017; 66: 1631–1644. 27267903 10.1136/gutjnl-2015-310691

[4] Cooper JA Parsons N Stinton C, et al. Risk-adjusted colorectal cancer screening using the FIT and routine screening data: development of a risk prediction model. Br J Cancer 2018; 118: 285–293. 29096402 10.1038/bjc.2017.375 PMC5785737

[5] Cooper JA Moss SM Smith S, et al. FIT for the future: a case for risk-based colorectal cancer screening using the faecal immunochemical test. Colorectal Dis 2016; 18: 650–653. 27135192 10.1111/codi.13365

[6] Cairns SR Scholefield JH Steele RJ, et al. Guidelines for colorectal cancer screening and surveillance in moderate and high risk groups (update from 2002). Gut 2010; 59: 666–689. 20427401 10.1136/gut.2009.179804

[7] https://www.gov.uk/government/collections/english-indices-of-deprivation (accessed 30 September 2020).

[8] Fraser CG Benton SC. Detection capability of quantitative faecal immunochemical tests for haemoglobin (FIT) and reporting of low faecal haemoglobin concentrations. Clin Chem Lab Med 2019; 57: 611–616. 29995629 10.1515/cclm-2018-0464

[9] Amemiya T. Regression analysis when the dependent variable is truncated normal. Econometrica 1973; 41: 997–1016.

[10] Clark GRC Strachan JA McPherson A, et al. Faecal haemoglobin distributions by sex, age, deprivation and geographical region: consequences for colorectal cancer screening strategies. Clin Chem Lab Med 2020; 58: 2073–2080. DOI: 10.1515/cclm-2020-0268. 10.1515/cclm-2020-0268 32324157

[11] Brenner H Qian J Werner S. Variation of diagnostic performance of fecal immunochemical testing for hemoglobin by sex and age: results from a large screening cohort. CLEP 2018; 10: 381–389. 10.2147/CLEP.S155548 PMC5896664 29670403

[12] Ribbing WH Blom J Hoijer J, et al. Fecal immunochemical test in cancer screening - colonoscopy outcome in FIT positives and negatives. Scand J Gastroenterol 2019; 54: 303–310. 30907196 10.1080/00365521.2019.1585569

[13] Digby J Fraser CG Carey FA, et al. Faecal haemoglobin concentration is related to severity of colorectal neoplasia. J Clin Pathol 2013; 66: 415–419. 23418340 10.1136/jclinpath-2013-201445

[14] Whyte S Thomas C Kearns B, et al. Optimising Bowel Cancer Screening Phase 1: Optimising the cost effectiveness of repeated FIT screening and screening strategies combining bowel scope and FIT screening. 2017. Report. ScHARR HEDs Discussion Papers. School of Health and Related Research (ScHARR), University of Sheffield, Sheffield.

[15] Digby J Fraser CG Carey FA, et al. Can the performance of a quantitative FIT-based colorectal cancer screening programme be enhanced by lowering the threshold and increasing the interval? Gut 2018; 67: 993–994. 28838973 10.1136/gutjnl-2017-314862

[16] van Roon AH Goede SL van Ballegooijen M, et al. Random comparison of repeated faecal immunochemical testing at different intervals for population-based colorectal cancer screening. Gut 2013; 62: 409–415. 22387523 10.1136/gutjnl-2011-301583

[17] Toes-Zoutendijk E Kooyker AI Dekker E, et al. Incidence of interval colorectal cancer after negative results from first-round fecal immunochemical screening tests, by cutoff value and participant sex and age. Clin Gastroenterol Hepatol 2020; 18: 1493–1500. 31442598 10.1016/j.cgh.2019.08.021

[18] van Roon AH Wilschut JA Hol L, et al. Diagnostic yield improves with collection of 2 samples in fecal immunochemical test screening without affecting attendance. Clin Gastroenterol Hepatol 2011; 9: 333–339. 21185397 10.1016/j.cgh.2010.12.012

[19] Niedermaier T Weigl K Hoffmeister M, et al. Diagnostic performance of flexible sigmoidoscopy combined with fecal immunochemical test in colorectal cancer screening: Meta-analysis and modeling. Eur J Epidemiol 2017; 32: 481–493. 28667446 10.1007/s10654-017-0279-2

[20] Haug U Grobbee EJ Lansdorp-Vogelaar I, et al. Immunochemical faecal occult blood testing to screen for colorectal cancer: can the screening interval be extended? Gut 2017; 66: 1262–1267. 27006184 10.1136/gutjnl-2015-310102

[21] Cong R. Truncated regression. Stata Tech Bull 1999; 52: 47–52.

[22] Ciatto S Martinelli F Castiglione G, et al. Association of FOBT-assessed faecal Hb content with colonic lesions detected in the florence screening programme. Br J Cancer 2007; 96: 218–221. 17211476 10.1038/sj.bjc.6603534 PMC2359986

