# Supplementary material for: Faecal immunochemical testing in bowel cancer screening: Estimating outcomes for different diagnostic policies
Source: J Med Screen. 2020 Dec 20;28(3):277–85. doi: 10.1177/0969141320980501 (PMC8366184; doi:10.1177/0969141320980501)
Supplement: sj-pdf-1-msc-10.1177_0969141320980501 - Supplemental material for Faecal immunochemical testing in bowel cancer screening: Estimating outcomes for different diagnostic policies [file sj-pdf-1-msc-10.1177_0969141320980501.pdf]

Supplementary materials

Table S1: Regression results from the final truncated model of natural logarithm of f-Hb on age, sex and pathology outcome, using 1,825 complete cases.

Variable	Coefficient	S.E	95% CI	p-value†
**Age (in years)**	-0.03	0.022	-0.073, 0.015	0.203
**Sex**				
**Female**	Baseline	-	-	-
**Male**	0.48	0.198	0.090, 0.865	0.016
**Pathology**				
**No abnormality**	Baseline	-	-	-
**CRC**	3.08	0.484	2.134, 4.029	<0.001
**HRA**	1.53	0.380	0.788, 2.278	<0.001
**IRA**	0.97	0.364	0.255, 1.682	0.008
**LRA**	-0.06	0.335	-0.717, 0.595	0.855
**Other abnormality**	0.09	0.325	-0.550, 0.723	0.791
**Intercept**	3.05	1.53	0.057, 6.041	0.046

S.E: standard error; 95% CI: confidence interval; CRC: colorectal cancer;

HRA, IRA and LRA are high-, intermediate- and low-risk adenomas respectively.

† Based on Wald test.

Table S2: Frequency of different pathologies by f-Hb categories from the FIT pilot study and estimates from the univariable regression.

f-Hb category	CRC	Change†	HRA	Change†	IRA	Change†	LRA	Change†	No/Other abnormality*	Change†	Total
**<4**	*4*	.	*33*	.	*37*	.	*555*	.	20,804	.	21,433
**4-9**	*5*	.	*42*	.	*56*	.	*299*	.	1,980	.	2,382
**10-19**	*7*	.	*45*	.	*62*	.	*217*	.	959	.	1,290
**20-39**	*10*	-2	*52*	-4	*72*	+1	*180*	-6	309	+11	623
**40-79**	*12*	+1	*52*	+8	*69*	0	*130*	+19	269	-28	532
**80-119**	*8*	+2	*26*	-2	*33*	+5	*52*	-4	71	-1	190
**120-149**	*4*	-1	*14*	0	*16*	-1	*23*	-3	23	+5	80
**150-179**	*3*	+1	*9*	+2	*12*	-6	*16*	+2	21	+1	61
**180+**	*37*	-1	*63*	-4	*59*	+1	*69*	-8	111	+12	339
**Total**	*90*	.	*336*	.	*416*	.	*1,541*		24,547	.	27,238

f-Hb: faecal haemoglobin concentration (μg/g); CRC: colorectal cancer; HRA, IRA and LRA are high-, intermediate- and low-risk adenomas respectively.

Frequencies for CRC, HRA, IRA and LRA (in italics) are estimated from the univariable regression.

* Frequency for 'other/normal' column is subtracted from observed total for each f-Hb category.

† Change is the difference between estimated and observed frequencies, i.e. for f-Hb 20-39 μg/g, 10 CRC are estimated; however we observed 8 CRC in the study.

Table S3: Estimated prevalence and cumulative prevalence of CRC and adenomas by f-Hb categories.

	Prevalence (1 in X)					Cumulative Prevalence (1 in X)				
**f-Hb category**	**CRC**	**HRA**	**IRA**	**LRA**	**HR+**	**Cancer**	**HRA**	**IRA**	**LRA**	**HR+**
**<4**	5,358	649	579	39	579	303	81	65	18	64
**4-9**	476	57	43	8	51	68	19	15	6	15
**10-19**	184	29	21	6	25	42	13	11	5	10
**20-39**	71	14	10	4	11	29	10	8	5	7
**40-79**	52	12	9	5	10	22	9	8	5	6
**80-119**	28	9	7	4	7	15	7	7	5	5
**120-149**	23	7	6	4	5	13	7	7	5	4
**150-179**	24	8	6	5	6	12	7	7	6	4
**180+**	11	7	7	6	4	11	7	7	6	4

CRC: colorectal cancer; f-Hb: faecal haemoglobin concentration (μg/g);

HRA, IRA and LRA are high-, intermediate- and low-risk adenomas respectively.

HR+ are colorectal cancers and high risk adenomas combined.

Table S4: Estimated sensitivity of colorectal abnormalities by f-Hb threshold.

	Sensitivity				
**Threshold, μg/g**	**CRC**	**HRA**	**IRA**	**LRA**	**HR+**
**4**	95.6%	90.2%	91.1%	64.0%	91.3%
**10**	90.0%	77.7%	77.6%	44.6%	80.3%
**20**	82.2%	64.3%	62.7%	30.5%	68.1%
**40**	71.1%	48.8%	45.4%	18.8%	53.5%
**80**	57.8%	33.3%	28.8%	10.4%	38.5%
**120**	48.9%	25.6%	20.9%	7.0%	30.5%
**150**	44.4%	21.4%	17.1%	5.5%	26.3%
**180**	41.1%	18.8%	14.2%	4.5%	23.5%

CRC: colorectal cancer; f-Hb: faecal haemoglobin concentration (μg/g);

HRA, IRA and LRA are high-, intermediate- and low-risk adenomas respectively.

HR+ are colorectal cancers and high risk adenomas combined.

Table S5 Estimated thresholds and observed percentage positives above each threshold. correspond to required sensitivities for colorectal cancer and high-risk adenomas.

**Abnormality**	Required sensitivity, %	Threshold in μg/g	Observed positives[†], %
	50.0	111	2.2
	60.0	68	3.4
**Colorectal cancer**	70.0	40	5.2
	80.0	22	7.5
	90.0	9	13.3
	50.0	36	5.7
	60.0	23	7.4
**High-risk adenoma**	70.0	14	9.9
	80.0	8	14.2
	90.0	4	21.3

[†] % of participants who had faecal haemoglobin concentration above each threshold and requires a further investigation in the FIT pilot study.
